# Different tumorigenicity and distinct metastasis and gene signature between orthotopic and subcutaneous neuroblastoma xenografted mice

**DOI:** 10.18632/aging.203913

**Published:** 2022-02-23

**Authors:** Rui Han, Wenjie Zhao, Xu Gu, Xue Gao, Yong-Guang Yang, Xiaoling Zhang

**Affiliations:** 1 Key Laboratory of Organ Regeneration and Transplantation of Ministry of Education, The First Hospital of Jilin University, Changchun, China; 2 National-Local Joint Engineering Laboratory of Animal Models for Human Diseases, Changchun, China; 3 International Center of Future Science, Jilin University, Changchun, China

**Keywords:** neuroblastoma, orthotopic human NB CDX model, subcutaneous human NB CDX model, metastasis, disease model

## Abstract

Patient-derived (PDX) and cell-derived (CDX) xenograft models are widely used in preclinical studies of human neuroblastoma. In this study, we constructed orthotopic and subcutaneous neuroblastoma CDX models by injecting human neuroblastoma cells into the adrenal gland and the flanks of immunodeficient mice, respectively. The tumorigenesis, metastasis and response to chemotherapy for the two models were also compared. Our results indicated that orthotopic tumor mice showed significantly faster tumor growth than that of subcutaneous mice. Importantly, the expression of *PHOX2B* and *GAB2* was dramatically increased in the tumors of orthotopic CDX mice. Furthermore, orthotopic CDX mice developed multiple organ metastasis resembling that of neuroblastoma patients, while metastasis occurred predominantly in lung in subcutaneous CDX mice. Moreover, the two CDX models showed comparable response to cyclophosphamide treatment. Our results suggest that orthotopic CDX mice are superior to subcutaneous CDX mice as a preclinical model to study human neuroblastoma.

## INTRODUCTION

Neuroblastoma (NB), which arises from the neural crest cells of the peripheral sympathetic nervous system, is frequently observed in adrenal glands [1]. NB is the most common extracranial malignant solid tumor in children and contributes to about 15% childhood cancer mortality [2, 3]. Therefore, NB is an important cause of tumor deaths in children aged 1 to 4 years [4]. Many high-risk NB patients show no response to conventional treatments. Furthermore, NB patients easily develop resistance to chemotherapy treatments that are currently used. Therefore, it is of importance to fully understand the underlying mechanisms for the development, metastasis and recurrence of high-risk NB and subsequently develop new therapeutic strategies to improve the survival rate of patients with high-risk neuroblastoma.

NB is characterized by early metastasis to the bone, lung, brain, and bone marrow [5]. However, results from preclinical studies do not closely mimic those from human clinical trials which may be an important reason for late-stage human clinical trial failures. It is well-known that xenograft mice models, including subcutaneous xenograft (SX) and orthotopic xenograft (OX) models, which are widely used to investigate tumorigenesis, development of anti-cancer drugs and exploration of key molecular mechanisms for tumors [6]. Therefore, improved mouse models that better reflect human disease are of significant importance in studying tumorigenesis and developing personalized anti-cancer drugs. Although, the subcutaneous transplantation models are used in the verification of cellular based results and pharmaceutical trials, the rarely occurred local infiltration and distant metastasis, the failure to fully simulate the natural growth environment and biological behavior of some tumors in the subcutaneous xenograft mice models limited their wide application in cancer research [7]. It was reported that in gallbladder cancer xenograft mice models, the ascites generation, lymph node and liver metastasis can only be detected in orthotopic models but not in subcutaneous models [8]. However, the orthotopic models, which are highly advocated by researchers, can simulate the tumor growing environment and enable them to maintain the features of the primary tumor, thus being more patient-like models [9]. However, the operation of this model is relatively complicated and time-consuming. Therefore, the NB orthotopic xenograft models were rarely established due to the smallness of the adrenal glands and the fragility of the kidneys of the mice.

Here, we successfully established orthotopic xenograft model and subcutaneous xenograft model of NB using the SH-SY5Y cell line, and compared the two animal models in terms of tumor growth, metastasis, gene expression and response to cyclophosphamide treatment. Our results will help to select appropriate xenograft models according to experimental purposes and provide precise study of tumorigenesis and development of personalized medicine for cancer patients.

## METHODS

### Cells and reagents

Human NB SH-SY5Y cells were purchased from Eallbio Life Sciences (Beijing, China) and cultured in RPMI 1640 medium containing 10% FBS (GIBCO), 1% penicillin streptomycin mixture (sigma Aldrich) and 1% glutamine (sigma Aldrich) at 37°C, 5% CO_2_.

The SH-SY5Y-Luc cell line stably expressing firefly luciferase was generated by Lenti-virus infection. The lentiviral package with firefly luciferase (PLVX-Luc-P2A-dsRed-Monomer-N1) was conducted in HEK293T cells by pMD2.G and psPAX2 package system. Briefly, PLVX-Luc-P2A-dsRed-Monomer-N1, pMD2.G and psPAX2 were mixed in Opti-MEM and were transfected into the HEK293T cells by Lipo2000. Six hours after transfection, the DMEM was replaced by fresh DMEM. The cell culture medium containing the virus was collected at 24 and 48 hours after transfection. 3 mL of virus solution was added to the SH-SY5Y cells with confluence of 60–70% and after 6 hours incubation, 3 mL of fresh RPMI 1640 medium was added into the cell. After 24 hours of infection, the medium was replaced with fresh RPMI 1640 medium. dsRed positive cells were sorted by FACS and subjected to extended culture to establish SH-SY5Y-Luc cell line stably expressing firefly luciferase.

### Animals

NOD-Prkdcem26Cd52Il2rgem26Cd22/Nju (NOD/SCID IL2rg−/− mice, NCG) mice (5–7 weeks) were purchased from Nanjing University-Nanjing Institute of Biomedicine. Mice were housed in SPF environment for one week before use. All animals received care in compliance with the guidelines outlined in the Guide for the Care and Use of Laboratory Animals. All procedures were approved by the Institutional Animal Care and Use Committee (IACUC) of the First Hospital of Jilin University.

### Establishment of orthotopic human NB CDX model

**Figure 1:**
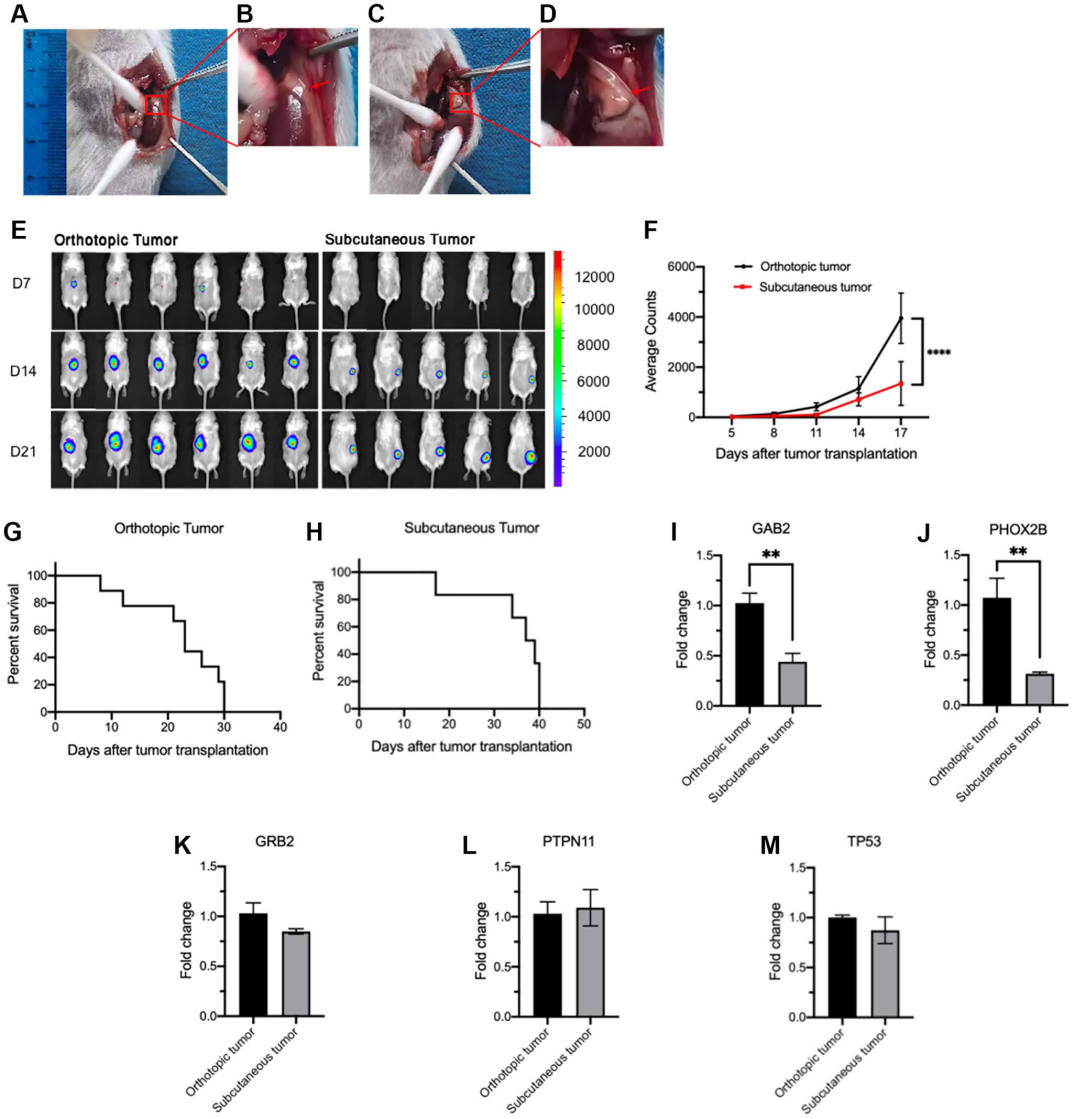
**Comparison of tumorigenesis and gene expression between orthotopic and subcutaneous CDX models.** (**A**–**D**) Illustration of orthotopic implantation. (**A**) Exposure of adrenal gland. (**B**) Local magnification of Figure 1A. (**C**) The expanded adrenal gland which is filled with tumor cells (20 μL). (**D**) Local magnification of Figure 1C. (**E**, **F**) Tumor growth measured by bioluminescence signals using *in vivo* imaging system. Representative bioluminescence images at the indicated time points (**E**) and tumor growth curves (**F**) (orthotopic, *n* = 6; subcutaneous, *n* = 5). (**G**, **H**) Survival curves of orthotopic (**G**, *n* = 8) and subcutaneous CDX mice (**H**, *n* = 8). (**I**–**M**) Relative expression of *GAB2* (**I**), *PHOX2B* (**J**), *GRB2* (**K**), *PTPN11* (**L**) and *TP53* (**M**) in orthotopic (*n* = 3) and subcutaneous tumor cells (*n* = 3). Data were normalized to the levels of orthotopic CDX mouse group. Data are presented as the mean ± SEM. ^**^P < 0.01. ^****^P < 0.0001.

Intraperitoneal injection of 0.05 g/mL tribromoethanol (35 mg/kg) to anesthetize the NCG mice (eight mice per group), fix them in the prone position on the surgical drape, prepare the skin on the left side of the back for disinfection, and gently lift the skin on the lower edge of the left rib with ophthalmic forceps. Cut the skin with ophthalmic scissors, and expand the wound to 1 cm to fully expose the muscle layer. Lift up the external oblique muscle above the spleen and create an incision. A sterile cotton swab was used to help to expose the left kidney and adrenal gland (Figure 1A and 1B). 1 × 10^5^ indicated cells were suspended in 20 μL PBS and were slowly injected into adrenal gland by 1 mL insulin syringe. The successful injection was confirmed by an expanded fascia between the kidney and adrenal gland (Figure 1C and 1D). After the injection is completed, withdraw the needle slowly and hold the cotton swab against the needle insertion position for 1 minute to prevent leakage. From the day of surgery, gentamicin sulfate water (80 mg gentamicin sulfate was diluted in 250 mL drinking water) was added for a week to prevent postoperative infection.

### Establishment of subcutaneous human NB CDX model

Intraperitoneal injection of 0.05 g/mL tribromoethanol (35 mg/kg) to anesthetize the NCG mice (eight mice per group), fix them in the prone position on the surgical drape, prepare the skin on the right side of the back for disinfection, lift the skin on the right back with ophthalmic forceps and make it down to form a gap. 1 × 10^5^ indicated cells were suspended in 100 μL PBS and were injected under the skin with 1 mL insulin syringe. The mice were placed on the electric blanket until they wake up.

### Cyclophosphamide (CTX) treatment

The secondary orthotopic CDX mice were constructed by implanting with tumor cells from primary orthotopic (Orth→Orth) or subcutaneous (Subq→Orth) CDX mice. The number of mice used in each group was presented in each figure legend. For the CTX treatment assay, xenografted mice were received an intraperitoneal injection of saline (control) or CTX (20 mg/kg) from day 4 to day 8 after tumor cells implantation. The bodyweight and tumor growth are measured every four days.

### Measurement of mouse body weight, tumor growth and mice survival

After the models are constructed, the tumor growth was observed and recorded by small animal phase forming system at indicated time. Before imaging, D-fluorescein potassium salt (15 mg/kg) was injected intraperitoneally for *in vivo* imaging. After 7–8 minutes, the anesthetized tumor bearing mice were put on the lift table of camera dark box, and the fluorescence signals were taken at 0.1 s, 0.5 s, 1 s, 5 s, 10 s and 20 s. According to the measured fluorescence intensity, the growth curve of transplanted tumor was drawn.

### Quantitative RT-PCR

Real-time PCR was used to measure the expression of indicated genes. According to the method provided by Takara company, PrimeScript RT region kit with gDNA Eraser (Perfect Real Time) was used for reverse transcription. The sequences of used primers are listed in Table 1. RT-PCR was performed using ChamQ Universal SYBR qPCR Master Mix (Vazyem China). RT-PCR reactions were performed in triplicate using ABI Prism Sequence Detection System.

**Table 1:** Primer sequence used in the experiments.

**Number**	**Genes**	**Primers**
1	*GAB2*-F	GTTCTATGTCCCGCAGGAATATC
	*GAB2*-R	CCCTGTGTCAAACCACATGC
2	*PHOX2B*-F	AACCCGATAAGGACCACTTTTG
	*PHOX2B*-R	AGAGTTTGTAAGGAACTGCGG
3	*GRB2*-F	CTGGGTGGTGAAGTTCAATTCT
	*GRB2*-R	GTTCTATGTCCCGCAGGAATATC
4	*PTPN11*-F	GAACTGTGCAGATCCTACCTCT
	*PTPN11*-R	TCTGGCTCTCTCGTACAAGAAA
5	*TP53*-F	CAGCACATGACGGAGGTTGT
	*TP53*-R	TCATCCAAATACTCCACACGC

### Statistical analysis

The GraphPad Prism version 8.0 was used to perform statistical analysis. A two-sided unpaired *t*-test was used to evaluate statistically significant differences between any two groups, whereas multiple group comparisons were performed by 2-way ANOVA. Results were expressed as the mean ± standard error of mean (SEM). Survival time differences were plotted using Kaplan-Meier curves and analyzed using the log-rank test. All *P*-values were considered significant when *P <* 0.05.

## RESULTS

### Orthotopic human NB CDX model showed faster tumorigenesis and higher mortality than subcutaneous human NB CDX model

Orthotopic and subcutaneous human NB CDX models were established by implanting 1 × 10^5^ tumor cells into the adrenal gland and the flanks of NCG mice, respectively (Figure 1A–1D). Bioluminescence image was detected and bioluminescence intensity was analyzed every 4 days to monitor the tumorigenesis and tumor growth. These results indicated that the orthotopic model showed significantly faster tumor growth than the subcutaneous model (Figure 1E, 1F). Furthermore, orthotopic xenograft mice exhibited a shorter survival time around 30 days (Figure 1G), however, average survival time for subcutaneous xenograft mice is around 40 days (Figure 1H). The expression of *GAB2*, *PHOX2B*, *GRB2*, *PTPN11* and *TP53* in tumors was detected by RT-PCR and the mRNA level of *GAB2* and *PHOX2B* was significantly higher in orthotopic CDX mice tumors than that in subcutaneous CDX mice tumors (Figure 1I, 1J). There was no significant difference in the expression of *GRB2*, *PTPN11* and *TP53* between the two groups (Figure 1K–1M).

### Distinct metastatic patterns between orthotopic and subcutaneous human NB CDX models

**Figure 2:**
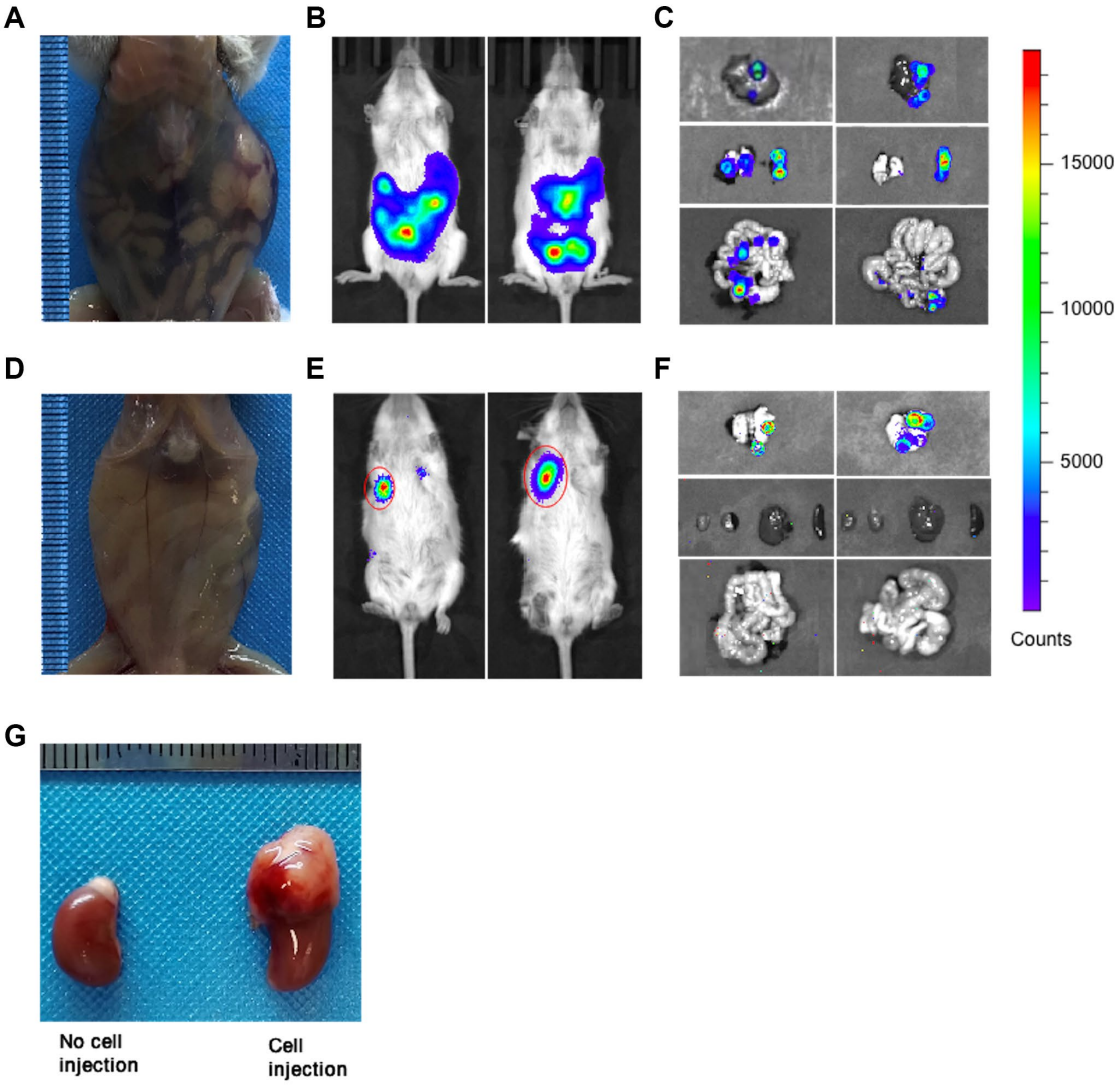
**Tumor metastasis in orthotopic and subcutaneous CDX mice.** (**A**) Representative image of orthotopic CDX mice was taken at day 14 after cell implantation (Mice used, *n* = 6, Representative data used, *n* = 1). (**B**) Representative bioluminescence images of tumors in orthotopic CDX mice (Mice used, *n* = 6, Representative data used, *n* = 2). (**C**) Representative bioluminescence images for the metastatic liver, lung, spleen and intestine in orthotopic CDX mice (Mice used, *n* = 6, Representative data used, *n* = 2). (**D**) Representative image of subcutaneous CDX mice were taken at day 21 after cell implantation (Mice used, *n* = 5, Representative data used, *n* = 1). (**E**) Representative bioluminescence images of tumors in subcutaneous CDX mice (Mice used, *n* = 5, Representative data used, *n* = 2). (**F**) Representative bioluminescence images for the metastatic liver, lung, spleen and intestine in subcutaneous CDX mice (Mice used, *n* = 5, Representative data used, *n* = 2). (**G**) Representative images for the kidneys and the adrenal glands were injected with or without cells (two kidneys from one CDX mouse). Left: Kidney without cell injection. Right: Kidney with cell injection.

Tumor metastasis in CDX mice was measured by bioluminescence signals using *in vivo* imaging system every 7 days (prone position). All orthotopic xenograft mice showed abdominal swelling and bloody ascites before death (Figure 2A), while ascites was not found in subcutaneous CDX mice until euthanasia due to excessive tumor burden (Figure 2D). All orthotopic CDX mice showed tumor metastasis in multiple organs from day 14 after implantation (Figure 2A, 2C), but the lung metastasis in the subcutaneous CDX mice was found at day 21 after implantation (Figure 2E, 2F). All orthotopic CDX mice we examined showed tumor metastasis in liver, spleen and intestine, and some of these mice also developed lung metastasis at later stage (Figure 2B, 2C). However, we did not observe abnormal shape and metastasis of kidney in orthotopic models (Figure 2G) at day 21 even until death. In contrast, subcutaneous CDX mice only showed lung metastasis (Figure 2E, 2F).

### Both orthotopic and subcutaneous CDX models are comparably responsive to cyclophosphamide chemotherapy

**Figure 3:**
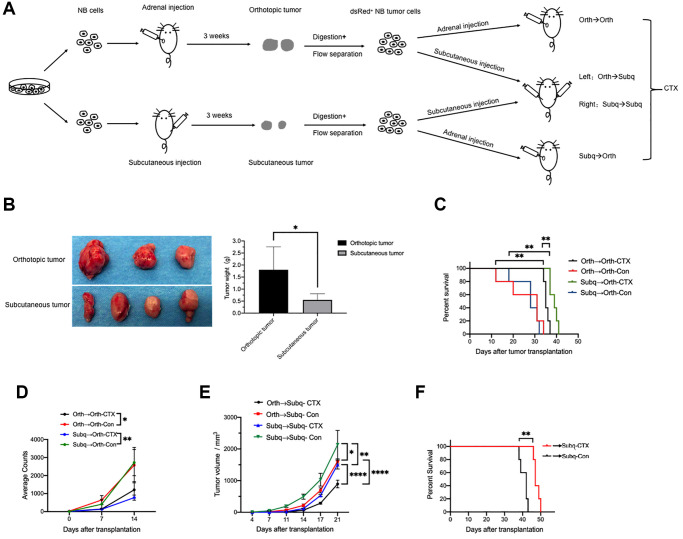
**Cyclophosphamide (CTX) suppresses tumor growth in both orthotopic and subcutaneous CDX models**. (**A**) Schematic of the experimental design for tumor cells implantation and CTX treatment. (**B**) Images and weight of dissected tumors from primary orthotopic (*n* = 3) and subcutaneous (*n* = 4) xenograft mice. The tumors were then digested and inoculated into NCG mice to construct the secondary orthotopic or subcutaneous CDX mice as indicated. (**C**) Survival curves of secondary orthotopic CDX mice which were implanted with tumor cells from primary orthotopic (Orth→Orth) or subcutaneous (Subq→Orth) CDX mice were treated with CTX or saline (control) (*n* = 5). (**D**) The average counts of bioluminescence signals of tumors in the secondary Orth→Orth (*n* = 4) and Subq→Orth (*n* = 3) CDX mice treated with or without CTX were measured at indicated time points. (**E**) *In vivo* tumor growth was monitored at the indicated time points in (**C**) (*n* = 5). (**F**) Survival curves of secondary Orth→Subq and Subq→Subq CDX mice treated with or without CTX (*n* = 5). Data are presented as the mean ± SEM. ^*^P < 0.05; ^**^P < 0.01; ^****^P < 0.0001.

Tumors from the orthotopic and subcutaneous CDX mice were dissected and digested at day 21 post-implantation of tumor cells to construct secondary orthotopic and subcutaneous xenograft mice which subsequently were treated with cyclophosphamide (CTX, 20 mg/kg; i.p.) or saline (Figure 3A). Consistently, the tumors isolated from primary orthotopic CDX mice were significantly larger than those in primary subcutaneous CDX mice (Figure 3B), and these tumors were digested and used to construct secondary orthotopic and subcutaneous xenograft mice. However, there was no significant difference in survival time between secondary Orth→Orth CDX mice and Subq→Orth CDX mice which were all responsive to CTX treatment (Figure 3C, 3D). Interestingly, secondary Subq→Subq CDX mice showed significantly faster tumor growth than Orth→Subq CDX mice, while both groups were comparably responsive to CTX treatment (Figure 3E), and the CTX treatment dramatically extended the survival time of Subq→Subq and Orth→Subq CDX mice (Figure 3F). These results indicate that, although orthotopic and subcutaneous CDX mice were highly different in tumorigenesis and metastasis, both models were comparably responsive to CTX treatment.

## DISCUSSION

Xenograft mice models including orthotopic and subcutaneous xenograft mice are widely used as preclinical models for understanding cancer biology and testing novel therapies. However, there are still some differences in recapitulating the characteristics of human tumorigenesis between the subcutaneous and orthotopic xenograft mice models which are fairly investigated in NB. In this study, we showed that orthotopic and subcutaneous human NB CDX models were highly different in tumorigenesis, metastasis and tumor-associated gene expression. The orthotopic CDX mice showed significantly faster tumor growth than subcutaneous CDX mice. In line with tumor growth, orthotopic tumors also showed higher expression of *GAB2*, *PHOX2B* genes than the subcutaneous tumor.

The interactions between tumor cells and the surrounding microenvironment of the host organ will affect the tumor growth, invasion or metastasis. The orthotopic xenograft model provides a similar grafting site and an equivalent or similar microenvironment for tumor cells as the primary site of tumors in human organ [8]. Tumor angiogenesis is essential for tumor cell proliferation, invasion and metastasis. Adrenal gland might be helpful to generate the new blood vessels in tumors by providing appropriate internal tumor surroundings [10]. Consistently, in one study, the angiogenesis, tumor growth and metastases in NB orthotopic and subcutaneous NB tumors were compared and the pattern of angiogenesis and metastases was distinctive for orthotopic and subcutaneous xenograft model. However, different from our findings, there was no difference in tumor growth between their orthotopic and subcutaneous NB tumors which may be caused by the different implantation site of tumor cells between their and our subcutaneous tumor models [11]. Therefore, the adrenal gland in our orthotopic xenograft model can provide a suitable microenvironment for SH-SY5Y cell growth. Consistently, we found the implanted SH-SY5Y cells grow faster in orthotopic CDX mice than that in subcutaneous CDX mice. More interestingly, the secondary orthotopic CDX mice (Orth→Orth) and subcutaneous (Subq→Subq) CDX mice showed a similar tumor growth pattern which indicated the importance of grafting site in supporting tumor growth. It was also reported that the microenvironment promotes the tumor invasion or metastasis [12]. In our study, tumor metastasis in spleen, liver and intestine occurred as early as day 14 after tumor cell implantation in orthotopic xenograft mice. However, subcutaneous CDX mice showed distinct patterns of tumor metastasis. The metastasis was not detected until day 21 after tumor implantation, and the metastasis detected at later time was exclusively in the lung tissue. In NB patients, early metastasis is much more common in the bone marrow, lymph nodes and liver [13, 14], while lung metastasis is considered to be a terminal event [15, 16]. Such variations of metastasis imply that the organ environment can influence the tumor growth and its potential of metastasis.

It was reported that *GAB2* plays important roles in neuroblastoma pathogenesis through promoting the cooperation of *SHP2/MYCN* [17]. Furthermore, the transcription factor *PHOX2B* is an immunohistochemical marker for neuroblastoma [18] and a predisposing gene to hereditary neuroblastic tumors [19]. A previous study demonstrated that high expression levels of *PHOX2B* promoted the growth of human neuroblastoma cells in xenograft model [20]. Therefore, according to the phenotypes we observed and the functions of these genes, we selected *GAB2*, *PHOX2B*, *GRB2*, *PTPN11* and *TP53* as candidates to measure their expression which may be the underlying molecular mechanisms for the difference of tumor growth and metastasis between the two xenograft models. Consistently, we found the orthotopic tumors showed a higher expression of *GAB2* and *PHOX2B* than that in subcutaneous tumors which might contribute to the faster growth and multi-organ metastasis of tumors in orthotopic CDX model.

Most children with intermediate-risk and high-risk neuroblastoma will need to have chemotherapy and cyclophosphamide is one of the commonly used drugs [21–24]. We intend to explore whether the environment influences the tumor cell characteristics and the subsequent drug sensitivity after long time growth in orthotopic and subcutaneous mice, therefore, we re-inoculated the tumor cells to different sites such as Ortho to Subq and Subq to Ortho. In the course of cyclophosphamide administration, the decrease of tumor volume and the significant extension of survival period were observed in the orthotopic and subcutaneous CDX model. Furthermore, because of the following reasons, 1) in the orthotopic CDX model, tumor grows much faster and the tumor size cannot be measured accurately due to the inner location, 2) the bioluminescence signals only partially but not accurately reflect tumor growth, 3) survival time could also reflect the degree of malignancy of tumor, 4) tumor growth in subcutaneous xenograft models is easy to measure, we mainly measured the survival time for the orthotopic CDX models and the tumor volume for the subcutaneous xenograft models. Importantly, both xenograft models showed comparable response to chemotherapy in terms of tumor growth which may partially explain the reason why subcutaneous CDX models are usually used to assess antitumor activity except for their high reproducibility, simple operation and ease of monitoring tumor growth. However, according to the published and our results, orthotopic model is better than subcutaneous CDX model in investigating the effect of drugs on the angiogenesis and metastases.

Altogether, the orthotopic and subcutaneous xenograft models we constructed in immunodeficient mice are two ideal models for the study of tumorigenesis and metastasis of neuroblastoma. Therefore, our study provided molecular rationale for researchers to choose suitable animal models for the pre-clinical investigation of new strategies to study and combat neuroblastoma according to their experimental purpose.
